# Long-term sport participation is associated with a selective auditory-musical performance profile

**DOI:** 10.3389/fpsyg.2026.1758520

**Published:** 2026-10-15

**Authors:** Bartłomiej Jacek Swebodziński, Kuba Korta, Jacek Łantowski

**Affiliations:** Institute of Psychology, Cardinal Stefan Wyszynski University in Warsaw, Warsaw, Poland

**Keywords:** long-term sport participation, *Montreal Battery of Evaluation of Amusia*, musical perception, perceptual-cognitive expertise, recognition memory, rhythm perception, temporal processing

## Abstract

Long-term sport participation has been associated with selective differences in temporal and perceptual-cognitive processing, but it remains unclear whether such differences extend to auditory-musical perception. We examined overall and component-level performance on the *Montreal Battery of Evaluation of Amusia* (MBEA) in 83 participants: 31 sport-trained participants and 52 psychology-student controls. Between-group differences were tested using ordinary least-squares regression with HC3 robust standard errors, adjusting for age and years of formal musical education; Holm correction was applied across the six MBEA subtests. Sport-trained participants obtained higher Total MBEA scores than controls (*B* = 15.34, *p* = 0.002). At the component level, they scored higher on the *a priori* temporal outcomes *Rhythm* (*B* = 2.87, Holm-adjusted *p* = 0.030) and *Meter* (*B* = 3.72, Holm-adjusted *p* = 0.012). An exploratory difference was also observed for *Memory* (*B* = 4.20, Holm-adjusted *p* = 0.002), whereas *Contour* did not remain significant after correction and no significant differences were found for *Scale* or *Interval*. Supplementary *d*′ models and their leave-one-out sensitivity analyses converged on the same pattern for *Rhythm*, *Meter*, and *Memory*. Within the sport-trained group, years of sport participation were not positively associated with Total MBEA performance after adjustment for age (*B* = −0.78, *p* = 0.108). These findings suggest that long-term sport participation is associated with a selective rather than generalized profile of auditory-musical differences, with the clearest group differences in temporal processing and an exploratory difference in recognition memory. The cross-sectional design does not permit causal attribution to sport training, and the observed profile may reflect training-related adaptations, pre-existing individual differences, or both.

## Introduction

Many forms of sustained sport participation place substantial demands on the rapid perception and use of temporally structured information to anticipate upcoming events and coordinate action under time pressure. Evidence from sport expertise research supports the importance of these processes for skilled performance. Across 142 studies, higher-skilled athletes showed better cognitive performance than lower-skilled athletes, with the largest differences observed for decision-making skills and sport-specific tasks (Kalén et al., 2021). More specifically, a meta-analysis of combat sports showed that expert athletes anticipated opponents’ actions more accurately and rapidly than non-experts (Zhang et al., 2022). The relevance of anticipatory processing to skilled performance is further supported by evidence that temporal-occlusion training can improve visual anticipation and that these gains transfer to representative laboratory and field tasks (Müller et al., 2024). Together, these findings indicate that temporal anticipation and the rapid adjustment of action are important components of skilled sport performance, raising the question of whether differences associated with long-term sport participation may also extend beyond the motor domain to perceptual processes that depend on temporal organization.

One perceptual domain in which temporal organization is particularly important is auditory rhythm processing. A systematic review and meta-analysis of 30 fMRI studies showed that musical rhythm engages a broad bilateral cortico-subcortical network involving auditory as well as motor-related regions, including the superior temporal cortex, supplementary motor area, putamen, and cerebellum (Kasdan et al., 2022). More specifically, beat perception has been associated with increased putamen activity and stronger functional coupling between the putamen, supplementary motor area, premotor cortex, and auditory cortex, particularly when the beat must be internally generated (Grahn and Rowe, 2009). Contemporary accounts therefore propose that beat anticipation may rely on action-like timing processes involving the supplementary motor area and dorsal striatum, even in the absence of overt movement (Cannon and Patel, 2021). Consistent with this view, MEG evidence indicates that sensorimotor cortex can encode temporal predictions directed toward auditory regions during passive listening, while overt rhythmic movement can further sharpen temporal selection of auditory information (Morillon and Baillet, 2017). Together, these findings provide a plausible framework for considering functional overlap between motor expertise and auditory temporal processing, without implying that auditory–motor integration itself was directly measured in the present study.

Evidence from athletic timing research suggests that expertise-related differences are selective rather than uniformly domain-general. In a synchronization–continuation paradigm, Braun Janzen et al. (2014) found that athletes were more precise than musicians and controls in continuous circle drawing, whereas musicians showed an advantage in discrete finger tapping, indicating that timing performance may depend on the nature of expertise and task demands. Similarly, Perrone et al. (2023) reported that expert swimmers were more accurate and consistent than both expert runners and non-athletes in time-reproduction and finger-tapping tasks, suggesting that temporal-processing profiles may also vary across sport disciplines. Recent findings further indicate that some cross-domain differences may extend beyond overtly sport-specific timing: expert swimmers outperformed amateur controls in a neutral visual duration-discrimination task across both silent and task-irrelevant rhythmic contexts, although the authors interpreted this pattern as evidence of bounded rather than unrestricted transfer (Liu et al., 2026). Evidence for analogous differences in auditory-musical perception is considerably more limited. In a recent study directly assessing auditory frequency and beat discrimination, musicians outperformed both athletes and controls, whereas athletes did not differ from controls on either measure (Kleber et al., 2025). Thus, although athletic expertise has been associated with selective advantages in temporal processing, it remains unclear whether such differences extend to musical perception and, if so, whether they are global or concentrated in particular auditory-musical components.

Musical perception is not a unitary faculty but comprises partially dissociable processing components, including melodic, temporal, and memory-related processes (Peretz and Coltheart, 2003). The *Montreal Battery of Evaluation of Amusia* (MBEA) provides separate behavioral measures of these components through three melodic subtests (*Scale*, *Contour*, and *Interval*), two temporal subtests (*Rhythm* and *Meter*), and a musical *Memory* subtest (Peretz et al., 2003). This component-based structure makes it possible to examine not only whether groups differ in overall auditory-musical performance, but also whether such differences are selectively expressed in particular aspects of musical processing. A previous master’s thesis using the full MBEA to compare musicians, athletes, dancers, and untrained controls found no statistically significant overall group differences, although descriptive profiles differed across expertise domains, with dancers showing higher average performance in temporal components and musicians in melodic components (Mano, 2022). Peer-reviewed evidence using the full MBEA to characterize auditory-musical profiles in athletes nevertheless remains scarce. Thus, the full MBEA provides a suitable framework for testing whether sport-related group differences are global or selectively concentrated in particular components of musical processing.

The present study examined whether long-term sport participation was associated with differences in auditory-musical performance assessed with the full MBEA. First, we hypothesized that sport-trained participants would obtain higher overall MBEA scores than controls (H1). Second, we hypothesized that sport-trained participants would show higher performance specifically on the *Rhythm* and *Meter* subtests, reflecting an expected difference in temporal aspects of musical processing (H2). Given previous evidence that the duration of sport participation may be associated with temporal performance (Jia et al., 2020), we further hypothesized that longer sport-participation tenure would be positively associated with overall MBEA performance within the sport-trained group (H3). Group differences in *Scale*, *Contour*, *Interval*, and *Memory* were examined exploratorily to characterize the broader profile of auditory-musical performance. Because age and formal musical education may contribute to individual differences in MBEA performance, the principal between-group analyses additionally accounted for age and years of formal musical education.

## Methods

### Participants

The study included two groups: a sport-trained group and a control group of psychology students. The sport-trained group was recruited from individuals with long-term involvement in sport, including those training in sports clubs and those with sport-related educational backgrounds at secondary or tertiary level. No formal minimum threshold for years of participation or competitive history was applied. The sport-trained group initially comprised 33 participants. Two individuals were excluded prior to the main analyses: one because of a history of head injury requiring neurosurgical intervention and one because they met the MBEA control-trial exclusion criterion, resulting in a final sample of 31 participants. Their mean age was 29.45 years (*SD* = 8.17; range = 20–53). Participants reported an average of 14.26 years of overall sport participation (*SD* = 5.60; range = 6–30) and 9.90 years of experience in their primary sport discipline (*SD* = 5.66; range = 2–20). Twenty-seven participants (87.1%) reported having taken part in sports competitions either currently or in the past, whereas four (12.9%) reported no history of competitive participation. At the time of assessment, 26 participants (83.9%) described their involvement as amateur, two (6.5%) as semi-professional, and three (9.7%) as professional. The sample represented a broad range of sport disciplines. The most frequently reported primary disciplines were running and swimming (*n* = 3 each), followed by dance, fitness, and triathlon (*n* = 2 each). Fifteen other disciplines were represented by one participant each, and four participants reported involvement in more than one sport discipline. Formal musical education was uncommon: three participants (9.7%) reported attending music school for a mean of 3.33 years (*SD* = 2.52; range = 1–6).

The control group initially comprised 67 psychology students and was not selected on the basis of athletic status. Fifteen participants were excluded prior to the main analyses: one because of moderate hearing loss, one because of a history of head injury requiring surgical intervention, and 13 because they met the MBEA control-trial exclusion criterion, yielding a final control sample of 52 participants. Their mean age was 25.96 years (*SD* = 10.07; range = 18–50). Six participants (11.5%) reported formal musical education, with a mean duration of 3.67 years (*SD* = 1.37; range = 2–5). No additional hearing loss or relevant neurological history was reported in the final sample.

### Measures

Musical perception was assessed using the full *Montreal Battery of Evaluation of Amusia* (MBEA; Peretz et al., 2003), a standardized battery designed to assess distinct components of musical perception and memory. The MBEA comprises six subtests: three melodic subtests (*Scale*, *Contour*, and *Interval*), two temporal subtests (*Rhythm* and *Meter*), and a *Memory* subtest. In the *Scale*, *Contour*, *Interval*, and *Rhythm* subtests, participants compare pairs of melodies and judge whether they are the same or different with respect to the dimension assessed. In the *Meter* subtest, participants classify melodies as having a duple, march-like meter or a triple, waltz-like meter, whereas the *Memory* subtest assesses recognition of melodies presented earlier in the battery.

Each subtest yields a score ranging from 0 to 30, and the total MBEA score is calculated as the sum of performance across the six subtests, with a maximum possible score of 180. Practice trials precede the scored items. Control trials used to verify task engagement are included in the *Scale*, *Contour*, *Interval*, and *Rhythm* subtests; the *Meter* subtest does not contain a corresponding control item. Because the changes presented in the control trials were highly salient, a single incorrect “same” response was treated as potentially accidental. Participants were excluded if they gave an incorrect “same” response on at least two control trials across the *Scale*, *Contour*, *Interval*, and *Rhythm* subtests, which was taken to indicate insufficient attention to the task. All auditory stimuli were digitally synthesized from notated musical material, and participants completed the full battery in the standard sequence of *Scale*, *Contour*, *Interval*, *Rhythm*, *Meter*, and *Memory*.

### Procedure

Data were collected remotely using the LimeSurvey platform. Participants were recruited through social media and snowball sampling, with respondents invited to share the study link within their professional and academic networks. Participation was voluntary. Before beginning the study, participants received information about the study aims, data-handling procedures, and their right to withdraw at any time, and provided informed consent electronically. The study was approved by the local ethics board and conducted in accordance with the Declaration of Helsinki.

Following consent, participants completed a demographic and background questionnaire, including age, formal musical education, and screening questions concerning hearing loss and history of head injury. Participants in the sport-trained group additionally reported their sport discipline(s), overall years of sport participation, years of experience in their primary sport discipline, history of competition participation, and current level of sport involvement. The MBEA was then administered in its standard sequence: *Scale*, *Contour*, *Interval*, *Rhythm*, *Meter*, and *Memory*. Testing was fully automated. Participants were instructed to complete the tasks in a quiet environment using headphones, and the entire session lasted approximately 60 min.

### Statistical analysis

Age and years of formal musical education were included as covariates in all between-group regression models because both age and prior musical training have been associated with MBEA performance (Moreno-Gómez et al., 2017). Ordinary least-squares regression models were used throughout, with HC3 heteroskedasticity-consistent standard errors to provide robust inference.

To test H1, overall MBEA performance was examined with total MBEA score as the dependent variable and group (sport-trained participants vs. controls) as the predictor of primary interest, adjusting for age and years of formal musical education.

To test H2 and characterize the component-level profile of MBEA performance, separate regression models were fitted for each of the six MBEA subtests (*Scale*, *Contour*, *Interval*, *Rhythm*, *Meter*, and *Memory*), again with group as the predictor of interest and age and years of formal musical education as covariates. *Rhythm* and *Meter* constituted the two *a priori* temporal outcomes specified in H2, whereas *Scale*, *Contour*, *Interval*, and *Memory* were examined exploratorily. To control the family-wise error rate across the six component-level group comparisons, *p* values for the group coefficient were adjusted using the Holm procedure.

To test H3, the association between sport-participation tenure and overall MBEA performance was examined within the sport-trained group (*n* = 31), with total MBEA score as the dependent variable and overall years of sport participation as the predictor, adjusting for age. An additional exploratory model examined the association between overall years of sport participation and *Rhythm* performance within the sport-trained group, also adjusting for age.

As supplementary analyses, perceptual sensitivity was quantified using signal detection theory. For each MBEA subtest, *d*′ was calculated from item-level hit and false-alarm rates, with 15 signal and 15 noise trials per subtest. For the *Scale*, *Contour*, *Interval*, and *Rhythm* subtests, “different” trials were coded as signal and “same” trials as noise; for *Meter*, triple (waltz-like) trials were coded as signal and duple (march-like) trials as noise; and for *Memory*, previously presented melodies were coded as signal and novel melodies as noise. A loglinear correction was applied, with hit and false-alarm rates calculated as *H* = (hits + 0.5)/(N_signal + 1) and *FA* = (false alarms + 0.5)/(N_noise + 1), respectively. Perceptual sensitivity was then calculated as *d*′ = *z*(H) − *z*(FA), following signal-detection approaches previously applied to MBEA performance (Pfeifer and Hamann, 2015). Separate regression models were fitted for the six subtest-level *d*′ indices, with group as the predictor of interest and age and years of formal musical education as covariates. The Holm procedure was applied separately across the six *d*′ models. To assess the stability of these supplementary findings, leave-one-out sensitivity analyses were performed by sequentially excluding each participant and refitting the adjusted models with the Holm correction.

No *a priori* power analysis was conducted. Statistical analyses were conducted using IBM SPSS Statistics (version 29) and Python. SPSS was used for the original data preparation and descriptive analyses, whereas the revised regression models with HC3 heteroskedasticity-consistent standard errors, Holm-adjusted *p* values, supplementary *d*′ analyses, and leave-one-out sensitivity analyses were conducted in Python. All statistical tests were two-tailed, with α = 0.05. Holm-adjusted *p* values were used for inference in the two families of six component-level comparisons.

## Results

### Descriptive statistics

The final sample comprised 83 participants, including 31 sport-trained participants and 52 psychology-student controls. Descriptive statistics for the Total MBEA score and the six MBEA subtests are presented in Table 1.

**Table 1:** Descriptive statistics for MBEA scores by group.

MBEA measure	Controls (*n* = 52), *M* (*SD*)	Sport-trained (*n* = 31), *M* (*SD*)
Total MBEA	134.27 (25.51)	150.00 (16.86)
Scale	23.35 (5.17)	24.97 (3.28)
Contour	22.62 (4.72)	24.84 (3.62)
Interval	21.44 (4.75)	22.68 (3.82)
Rhythm	23.31 (5.36)	26.10 (3.58)
Meter	20.02 (5.07)	23.94 (5.38)
Memory	23.54 (6.05)	27.48 (2.79)

### Overall MBEA performance

In the adjusted regression model, sport-trained participants had higher Total MBEA scores than controls, *B* = 15.34, HC3 *SE* = 4.80, *t* = 3.19, *p* = 0.002, 95% CI [5.78, 24.90]. The model explained 13.9% of the variance in Total MBEA scores (*R*^2^ = 0.139, adjusted *R*^2^ = 0.106).

### Temporal MBEA components

After adjustment for age and years of formal musical education, sport-trained participants obtained higher scores than controls on both *a priori* temporal outcomes, *Rhythm* and *Meter*. Both group effects remained statistically significant after Holm correction (Table 2).

**Table 2:** Adjusted group effects for the six MBEA subtests.

MBEA subtest	*B*	HC3 *SE*	95% CI	*t*	*p*	Holm-adjusted *p*
Scale	1.23	0.91	[−0.58, 3.04]	1.35	0.181	0.361
Contour	2.14	0.98	[0.19, 4.08]	2.18	0.032	0.096
Interval	1.18	0.96	[−0.73, 3.08]	1.23	0.222	0.361
Rhythm	2.87	1.05	[0.79, 4.96]	2.75	0.007	0.030
Meter	3.72	1.19	[1.35, 6.09]	3.13	0.002	0.012
Memory	4.20	1.11	[1.98, 6.42]	3.77	< 0.001	0.002

Each model included group, age, and years of formal musical education. Group was coded 0 = controls and 1 = sport-trained participants. HC3 heteroskedasticity-consistent standard errors are reported. Holm-adjusted *p* values refer to the family of six group coefficients. Rhythm and Meter were the a priori outcomes specified in H2; Scale, Contour, Interval, and Memory were exploratory.

### Exploratory component-level analyses

Among the exploratory MBEA outcomes, the group effect for *Memory* remained statistically significant after Holm correction. The group effect for *Contour* was statistically significant before correction but did not remain so after Holm adjustment. No statistically significant group effects were observed for *Scale* or *Interval* (Table 2).

### Sport-participation tenure and overall MBEA performance

Within the sport-trained group, overall years of sport participation were not significantly associated with Total MBEA scores after adjustment for age, *B* = −0.78, HC3 *SE* = 0.47, *t*(28) = −1.66, *p* = 0.108, 95% CI [−1.73, 0.18], *R*^2^ = 0.079.

### Exploratory association between sport-participation tenure and *Rhythm*

An exploratory age-adjusted analysis within the sport-trained group showed that overall years of sport participation were negatively associated with *Rhythm* scores, *B* = −0.31, HC3 *SE* = 0.09, *t*(28) = −3.42, *p* = 0.002, 95% CI [−0.50, −0.13], *R*^2^ = 0.172. This association is illustrated in Figure 1.

**Figure 1:**
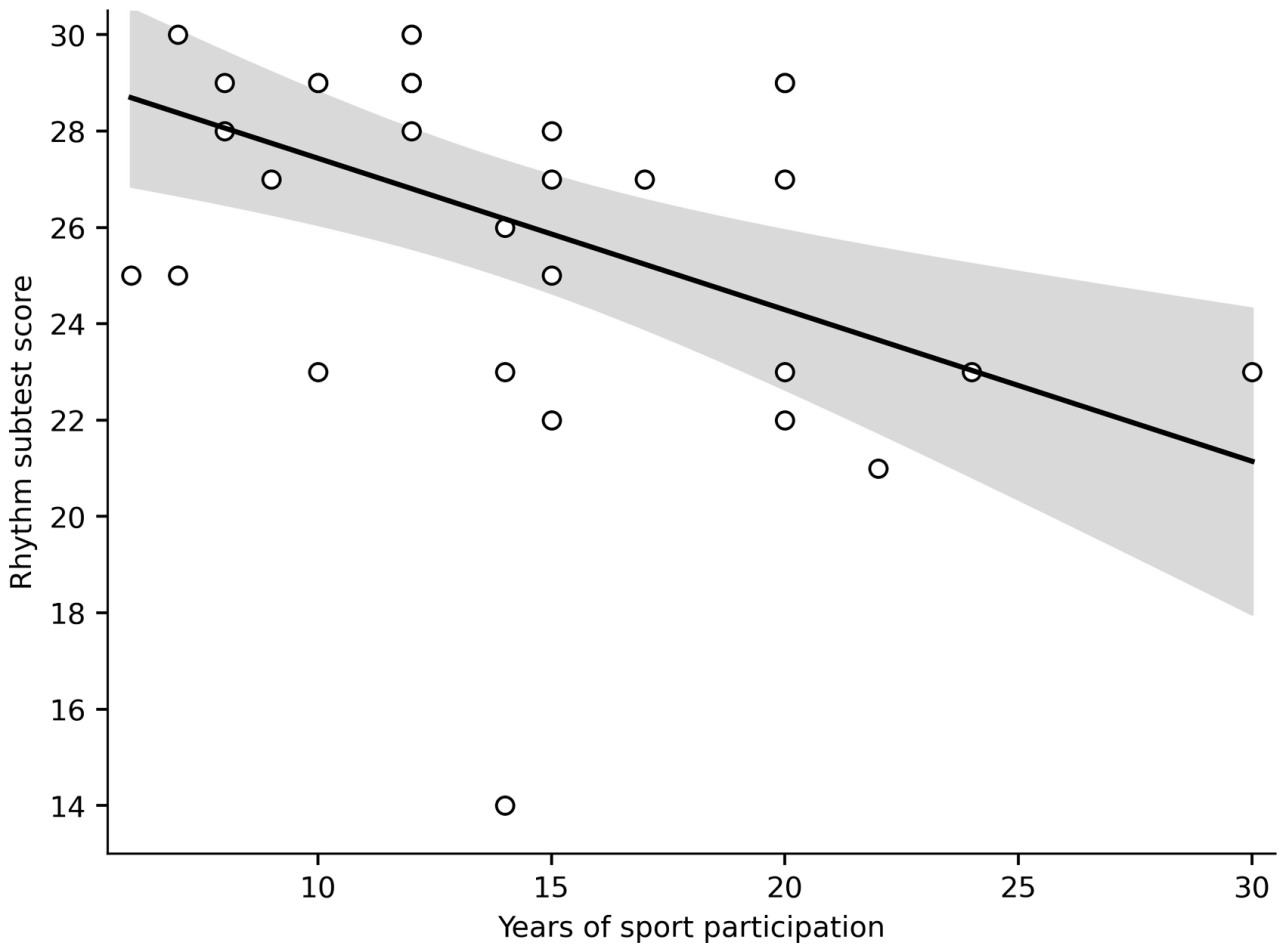
Association between overall years of sport participation and Rhythm scores in sport-trained participants. Points represent individual sport-trained participants (*n* = 31). The fitted line represents the age-adjusted association between overall years of sport participation and *Rhythm* scores, with age held at the sample mean. The shaded band represents the 95% confidence interval. HC3 heteroskedasticity-consistent standard errors were used for statistical inference.

### Supplementary *d*′ analyses

Supplementary analyses of perceptual sensitivity showed significant adjusted group effects for *Rhythm*, *Meter*, and *Memory* after Holm correction. The group effect for *Contour* was significant before correction but did not remain significant after Holm adjustment, whereas no significant group effects were observed for *Scale* or *Interval* (Table 3). Leave-one-out sensitivity analyses confirmed the stability of these effects: *Rhythm* (Holm-adjusted *p* range = 0.002–0.019), *Meter* (0.001–0.010), and *Memory* (<0.001–0.002) remained significant in all 83 iterations.

**Table 3:** Adjusted group effects for the six MBEA subtest d′ indices.

MBEA subtest	*B*	HC3 *SE*	95% CI	*p*	Holm-adjusted *p*
Scale	0.32	0.22	[−0.12, 0.76]	0.156	0.313
Contour	0.53	0.23	[0.08, 0.98]	0.023	0.068
Interval	0.20	0.19	[−0.18, 0.58]	0.304	0.313
Rhythm	0.75	0.24	[0.27, 1.22]	0.003	0.010
Meter	0.89	0.26	[0.37, 1.41]	0.001	0.005
Memory	1.02	0.26	[0.51, 1.53]	< 0.001	0.001

Each model included group, age, and years of formal musical education. Group was coded 0 = controls and 1 = sport-trained participants. HC3 heteroskedasticity-consistent standard errors are reported. Holm-adjusted p values refer to the family of six d′ group coefficients.

## Discussion

The present study examined whether long-term sport participation was associated with overall and component-level auditory-musical performance on the MBEA. Sport-trained participants showed higher overall MBEA performance than controls after accounting for age and formal musical education, in line with H1. At the component level, the hypothesized group differences emerged for *Rhythm* and *Meter*, consistent with H2, while an additional difference was observed for *Memory* in the exploratory analyses. By contrast, the group difference in *Contour* did not remain significant after correction for multiple comparisons, and no significant differences were found for *Scale* or *Interval*. Supplementary d′ analyses and leave-one-out sensitivity analyses converged on the same core pattern for *Rhythm*, *Meter*, and *Memory*, indicating that these component-level findings were not dependent on a single analytic approach or individual case. In contrast, sport-participation tenure was not positively associated with overall MBEA performance within the sport-trained group, and H3 was therefore not supported.

Although the higher total MBEA score indicates a group difference at the level of overall battery performance, it should not be interpreted as evidence of a generalized advantage in musical perception. Musical processing comprises partially dissociable components rather than a single unitary ability (Peretz and Coltheart, 2003), and the component-level findings showed that the overall group difference was not uniformly expressed across the MBEA subtests. Such selectivity is consistent with evidence that expertise-related perceptual differences depend on the specific demands of the task and training domain. Perrone et al. (2023), for example, reported differences in temporal performance even between athletes from different sport disciplines, while enhanced visual duration discrimination in expert swimmers has been interpreted as evidence of bounded rather than unrestricted cross-domain differences associated with sport expertise (Liu et al., 2026). Conversely, Kleber et al. (2025) found no advantage for athletes over controls in either auditory frequency or beat discrimination. Taken together, these findings suggest that the present total-score difference is more appropriately understood in terms of a selective component-level profile associated with long-term sport participation than as a broad enhancement of auditory-musical perception.

The group differences observed for *Rhythm* and *Meter* are particularly relevant because these two temporal components were specified *a priori* in H2. This pattern is consistent with evidence that long-term motor expertise can be associated with differences in temporal precision, although the form of such differences depends on the specific timing demands of the task. Braun Janzen et al. (2014), for example, found that athletes showed greater precision in continuous timed movement, whereas musicians showed an advantage in discrete timing, suggesting that temporal expertise is shaped by the characteristics of practiced action rather than reflecting a uniform domain-general ability. At the same time, expertise-related advantages appear to be strongly constrained by task and domain. In their meta-analysis of action anticipation using the temporal-occlusion paradigm, Song et al. (2025) found a substantial expert advantage within sport-specific contexts but no evidence that this advantage generalized to either familiar or unfamiliar sports. Accordingly, the present differences in *Rhythm* and *Meter* should not be interpreted as direct evidence that sport training transfers to auditory-musical timing. Rather, they indicate that long-term sport participation may be associated with individual differences in temporal processing that are also detectable in selected auditory-musical tasks. This possibility provides a rationale for considering whether sport-related and musical timing performance may draw, at least partly, on overlapping temporal-prediction and sensorimotor processes.

One possible framework for understanding the selective differences in *Rhythm* and *Meter* is the close functional coupling between auditory and motor systems in temporal processing. A meta-analysis of functional neuroimaging studies has shown that musical rhythm engages a distributed cortico-subcortical network including auditory cortex, the supplementary motor area, putamen, and cerebellum (Kasdan et al., 2022). More specifically, beat perception has been associated with putamen activity and increased functional coupling between the putamen, supplementary motor area, premotor cortex, and auditory cortex, particularly when temporal regularity must be internally represented (Grahn and Rowe, 2009). Consistent with these findings, theoretical accounts propose that beat anticipation may recruit action-like timing processes involving the supplementary motor area and dorsal striatum even in the absence of overt movement (Cannon and Patel, 2021). Experimental evidence further indicates that temporal predictions originating in sensorimotor cortex can shape auditory processing, while overt rhythmic movement can sharpen the temporal selection of auditory information (Morillon and Baillet, 2017). These findings provide a plausible basis for partial overlap between processes engaged by temporally structured motor activity and those supporting auditory-musical timing. However, because the present study did not directly assess auditory–motor synchronization, temporal prediction, or neural activity, this account should be regarded as a theoretical framework for interpreting the observed Rhythm and Meter differences rather than as evidence for a specific underlying mechanism.

The exploratory group difference observed in the *Memory* subtest warrants separate consideration. Unlike the preceding MBEA tasks, this subtest assesses incidental recognition of melodies encountered earlier in the battery, without advance instruction to memorize them (Peretz et al., 2003). Its persistence after correction for multiple comparisons, together with the convergent *d*′ and leave-one-out results, indicates that the finding was not readily attributable to response tendency or to a single influential observation in the present sample. One possibility is that it reflects efficiency in the incidental encoding and later recognition of structured sequential patterns rather than a domain-general memory advantage. This interpretation is compatible with research on perceptual-cognitive expertise in sport, in which pattern recognition, the use of contextual priors, and global processing have been identified as important components of skilled performance (DeCouto et al., 2024). Experimental findings further indicate that expert basketball players encode structural information from dynamic patterns more effectively than novices even when parts of that information fall outside the focus of attention (Gorman et al., 2013), while youth elite soccer players have shown more rapid early learning under implicit, but not explicit, sequential motor-learning conditions (Verburgh et al., 2016). These findings do not establish that such expertise-related processes generalize to unfamiliar auditory material, and the advantages documented in sport are typically closely tied to domain-relevant information. Nevertheless, they raise the possibility that the present *Memory* finding may reflect individual differences in the incidental extraction, encoding, or subsequent recognition of structured sequential information. Because the MBEA *Memory* task does not dissociate encoding, retention, and retrieval, nor directly assess implicit or statistical learning, this account should be regarded as a hypothesis generated by the exploratory finding and tested in future studies using independent auditory sequence-learning and recognition paradigms.

The absence of significant group differences in *Scale* and *Interval* further delineates the boundaries of the observed MBEA profile. These subtests depend primarily on aspects of melodic pitch organization: *Scale* requires sensitivity to tonal violations, whereas *Interval* requires discrimination of relatively fine-grained pitch relationships while preserving the melodic contour (Peretz et al., 2003). The lack of group differences on these measures therefore provides little support for a generalized advantage in pitch-based musical processing associated with long-term sport participation. This interpretation is consistent with recent evidence showing that trained musicians, but not athletes, outperform control participants in basic auditory frequency discrimination (Kleber et al., 2025), suggesting that enhanced pitch sensitivity may be more closely related to music-specific experience than to motor expertise per se. The *Contour* result deserves more cautious consideration. Melodic contour captures the directional shape of pitch changes and has often been conceptualized as a relatively global representation of melodic structure, in contrast to the more fine-grained interval code (Trainor et al., 2002). Although the uncorrected group difference in *Contour* may therefore be of interest in light of the exploratory findings concerning structural pattern recognition, it did not remain significant after correction for multiple comparisons and was not confirmed after correction in the *d*′ analyses. It should consequently be regarded as hypothesis-generating rather than as evidence for a reliable group difference. Taken together, the melodic findings reinforce the view that the present pattern is selective: the clearest group differences were observed in temporal components and, exploratorily, in recognition memory rather than in fine-grained pitch-based processing.

The absence of a positive association between sport-participation tenure and total MBEA performance provides an important qualification to the between-group findings. Contrary to H3, longer participation in sport was not associated with better overall auditory-musical performance within the sport-trained group, offering no support for a simple dose–response account in which accumulating years of sport participation are accompanied by progressively higher MBEA scores. Jia et al. (2020) reported an association between years of diving training and duration-perception effects for stimuli related to the athletes’ own sport, whereas the present hypothesis concerned performance in an unfamiliar auditory-musical domain. The findings are therefore not necessarily inconsistent and instead underscore the importance of task and domain specificity when considering associations with training history. An additional exploratory analysis unexpectedly indicated a negative association between sport-participation tenure and *rhythm* performance. Given its exploratory status and the relatively small and heterogeneous sport-trained sample, this finding should not be interpreted as evidence that prolonged sport participation adversely affects rhythmic perception. Rather, the tenure results as a whole argue against a straightforward linear relation between years of sport participation and the auditory-musical differences observed between groups. Years of participation are also an imprecise proxy for accumulated experience because they do not capture training intensity, quality, developmental timing, or the temporal demands of different sport disciplines.

The cross-sectional design of the present study does not allow the observed group differences to be attributed specifically to sport training. Research on the development of sport expertise emphasizes that skilled performance emerges from interactions among practice, environmental conditions, and pre-existing individual characteristics rather than from training exposure alone (Baker et al., 2003; Yan et al., 2016). This distinction is particularly important for perceptual-cognitive characteristics. In a large meta-analysis of cognitive functions and skills in sport, Kalén et al. (2021) noted that prospective evidence remains scarce, making it difficult to determine whether cognitive differences observed in experienced athletes primarily develop through practice or partly precede and facilitate sustained engagement in sport. A similar ambiguity applies to the present findings. Long-term sport participation may contribute to adaptations in temporal processing or pattern-based information processing, but individuals with pre-existing strengths in such processes may also be more likely to engage in, persist in, or become proficient at activities that repeatedly demand anticipation, sequencing, and rapid extraction of structure. These possibilities are not mutually exclusive and may operate reciprocally over development. The absence of a positive dose–response relationship with sport-participation tenure further cautions against privileging a simple cumulative-training explanation, although it does not exclude training-related effects that are nonlinear, depend on training quality or intensity, or emerge earlier in skill development. The present data therefore cannot distinguish selection effects from experience-dependent change.

Several additional limitations should be considered when interpreting these findings. First, the sport-trained group was relatively small and heterogeneous with respect to sport discipline, competitive history, and current level of involvement. Although this heterogeneity reflects the broader concept of long-term sport participation adopted in the present study, it limits the possibility of determining whether the observed auditory-musical profile is shared across different forms of sport experience or is more strongly associated with particular training demands. Second, the control group consisted of psychology students and was not selected on the basis of athletic status. Consequently, the groups should not be interpreted as representing a strict sport-trained versus non-active contrast, and unmeasured differences in current or previous physical activity may have influenced between-group differences. Third, remote administration limited experimental control. Although participants were instructed to complete the MBEA in a quiet environment using headphones, listening conditions could not be standardized to the degree possible in laboratory testing. In addition, no *a priori* power analysis was conducted, and the modest sport-trained sample limited the precision of estimates and the possibility of examining potentially informative subgroup effects. Finally, years of sport participation provided only a coarse measure of accumulated experience. The use of HC3 robust standard errors, correction for multiple comparisons, supplementary *d*′ analyses, and leave-one-out sensitivity analyses increases confidence that the principal component-level pattern is not an artifact of a single statistical specification or individual observation; however, these procedures cannot compensate for the sampling, measurement, and design constraints of the study.

Future studies should move beyond cross-sectional group comparisons to identify the processes underlying the selective auditory-musical differences observed here. Longitudinal designs beginning before or during the early stages of sustained sport participation would be particularly informative for distinguishing pre-existing individual differences from experience-dependent change. Such studies should characterize sport exposure more precisely than by training tenure alone, including training frequency and intensity, developmental timing, and the extent to which particular disciplines require temporal prediction, rhythmic coordination, or rapid sequencing of action. The temporal account suggested by the *Rhythm* and *Meter* findings could also be tested directly using independent measures of beat perception, duration discrimination, sensorimotor synchronization, and auditory temporal prediction, potentially combined with neurophysiological measures of auditory–motor coupling. A separate line of research is suggested by the exploratory *Memory* finding. Given the importance of pattern recognition, contextual information, and global processing in perceptual-cognitive expertise (DeCouto et al., 2024), future studies could examine whether long-term sport participation is associated with more efficient incidental encoding and recognition of novel structured sequences across auditory and non-auditory modalities. Experimental paradigms that manipulate sequence regularity and provide separate indices of encoding, learning, and subsequent recognition would help determine whether the present *Memory* difference reflects structural pattern extraction, retention, recognition processes, or some combination of these mechanisms. Replication in larger samples, with better characterized comparison groups and sufficient representation of different sport disciplines, would further allow these temporal and pattern-processing hypotheses to be tested separately rather than inferred from a heterogeneous sport-trained group.

In conclusion, long-term sport participation was associated with higher overall MBEA performance, with the clearest group differences observed in the *a priori* temporal components of *Rhythm* and *Meter* and an additional exploratory difference in *Memory*. The lack of Holm-corrected group differences across the melodic subtests, together with the absence of a positive association between sport-participation tenure and total MBEA performance, argues against interpreting this pattern as a generalized auditory-musical advantage or a simple cumulative effect of training duration. Rather, the findings are consistent with a selective profile of cross-domain differences that may involve temporal processing and, potentially, incidental encoding and recognition of structured sequential information. Because the present cross-sectional design cannot distinguish training-related change from pre-existing individual differences, these interpretations remain hypotheses to be tested directly in longitudinal and process-focused studies.

## Data Availability

The datasets presented in this study can be found in online repositories. The names of the repository/repositories and accession number(s) can be found at: https://doi.org/10.5281/zenodo.23134827.
